# Process evaluation and lessons learned from the formation of a multi-sector partnership: the Healing Experiences of Adversity among Latinos (HEALthy4You)

**DOI:** 10.3389/frhs.2025.1607665

**Published:** 2025-10-17

**Authors:** Clare Viglione, Amy Westermann, Job Godino, Kyung E. Rhee, Blanca Melendrez, Xin M. Tu, David L. Boyle, Michael Hogarth, Gregory A. Aarons, Noe C. Crespo, Pradeep Gidwani, Margarita Holguin, Cynthia Juarez, Deysi B. Merino-Gonzalez, Liliana Osorio, Herminia Ramirez, Micaela Smith, Alec Terrana, Gary S. Firestein, Eric Hekler

**Affiliations:** ^1^ Altman Clinical and Translational Research Institute, University of California, San Diego, La Jolla, CA, United States; ^2^ School of Medicine, University of California, San Diego, La Jolla, CA, United States; ^3^ Herbert Wertheim School of Public Health and Human Longevity Science, University of California, San Diego, La Jolla, CA, United States; ^4^ Laura Rodriguez Research Institute, Family Health Centers of San Diego, San Diego, CA, United States; ^5^ Department of Pediatrics, School of Medicine, University of California San Diego, La Jolla, CA, United States; ^6^ Center for Community Health, University of California, San Diego, San Diego, CA, United States; ^7^ Division of Biostatistics and Bioinformatics, Herbert Wertheim School of Public Health and Human Longevity Science, University of California, San Diego, La Jolla, CA, United States; ^8^ Department of Medicine, School of Medicine, University of California, San Diego, La Jolla, CA, United States; ^9^ Department of Psychiatry, School of Medicine, University of California San Diego, San Diego, CA, United States; ^10^ Dissemination and Implementation Science Center, Altman Clinical and Translational Research Institute, University of California San Diego, San Diego, CA, United States; ^11^ Child and Adolescent Services Research Center, San Diego, CA, United States; ^12^ School of Public Health, San Diego State University, San Diego, CA, United States; ^13^ Institute for Behavioral and Community Health, College of Health and Human Services, San Diego State University, San Diego, CA, United States; ^14^ American Academy of Pediatrics, California Chapter 3, San Diego, CA, United States; ^15^ Consulting Solutions, LLC, San Diego, CA, United States; ^16^ Vista Community Clinic, Vista, CA, United States; ^17^ YMCA of San Diego County, San Diego, CA, United States; ^18^ Department of Psychiatry & Behavioral Sciences, University of Washington, Seattle, WA, United States; ^19^ The Design Lab, University of California San Diego, La Jolla, CA, United States

**Keywords:** community-based participatory research, multi-sector partnership, implementation science, federally qualified health center, community co-creation, Hispanic/Latino community

## Abstract

The Healing Experiences of Adversity Among Latinos (HEALthy4You; H4Y) study was a multi-sector partnership between an academic research institution, a Federally Qualified Health Center (FQHC), and a multi-sector collective impact coalition focused on childhood obesity prevention. The goal of HEALthy4You was to develop community-centered and culturally appropriate precision interventions within FQHCs for Latino families to address predictors of adverse child experiences and treat childhood obesity. A multidisciplinary and multi-sector research, clinical, and community team (*N* = 29) was formed in September 2020 to co-design the study, which launched in June 2022. The team utilized a *co-creation* approach combined with the Exploration, Preparation, Implementation, and Sustainment framework to facilitate a collaborative design process. We conducted an internal and retrospective process evaluation in March 2023 to identify antecedents and situational factors associated with project formation, with a focus on understanding tensions and challenges with a broad partnership structure. We outline the team's co-creation process and describe internal challenges and pitfalls that emerged when developing the project. We sought to better understand the impact of differing perspectives, priorities, and goals between disciplines, sectors, and roles; differing approaches to evidence and evidence production; and team strategies to mitigate and manage competing pressures and priorities. This case report describes lessons learned, intending to share insights to support future development of best practices in project, partner, and team formation between researchers, clinicians, and community members. More specifically, these lessons could help inform community-led research endeavors between academic institutions, FQHCs, and community-based organizations (CBOs).

## 1 Introduction

There is increased recognition that addressing chronic disease and improving public health requires engagement with multiple sectors (e.g., community members, government, healthcare, and academia) to work in coordination, with participants from each sector doing their part to improve public health (1, 2). The community can play a critical role in defining goals, as well as cultivating “civic belonging” that is necessary for fostering effective accountability (1). The healthcare sector can provide primary, secondary, and tertiary medical care. Academia can provide evidence to develop guidelines and scientific interventions (3). Furthermore, government (e.g., public health) can play a critical role in fostering “vital conditions,” such as humane housing, reliable transportation, accessible food, meaningful work, and wealth that everyone needs to thrive. Thus, different sectors should come together and learn how to collaborate effectively to foster health for everyone, in every community, everywhere (4).

Multi-sector partnerships (MSPs) involve various sectors working together toward collective goals. These partnerships can systematically integrate diverse perspectives and resources to enhance the effectiveness and sustainability of research outcomes. While the process can be complex, leveraging the strengths and assets of different sectors empowers MSPs to address complex health and social issues more effectively (5, 6). One critically important MSP brings together communities, community-based organizations (CBOs), safety net healthcare systems, and, when research is needed, academics. Growing investment from funders, such as the Patient-Centered Outcomes Research Institute (PCORI), National Institutes of Health (NIH), and Robert Wood Johnson Foundation (RWJF), to support these collaborations indicates the value of these collaborations. For instance, the NIH's Community Partnerships to Advance Science for Society (ComPASS) Program exemplifies this by focusing on scaling community-led health equity interventions through MSPs to reduce health disparities (7). Despite this interest, there is limited data and insights provided about specific strategies for fostering effective MSPs. While elements from community-based participatory research (CBPR) have been applied to building research collaborations, there is little consensus on how to concretely employ CBPR for MSPs. Thus, there is a need for research about partnership processes and strategies to guide the field in appropriate expectations, funding models, and structures for operationalizing effective MSPs.

This report describes the establishment of an MSP of CBOs connected with a collective impact coalition, a federally qualified health center (FQHC), and a research university to co-create the HEALthy4You study in San Diego, CA, USA. The goal of HEALthy4You initiated in 2020 was to develop community-centered and culturally appropriate precision interventions for Latino families to address predictors of adverse child experiences (ACEs) and treat childhood obesity. The partnership culminated in a factorial trial at an FQHC to test family-centered, primary care interventions (i.e., parenting education, community health worker support, and nutritional counseling), coupled with a community-led research project focused on understanding the environmental and policy conditions that support or hinder child and family health and wellbeing. In this paper, we evaluate the multi-sector partnership formation and decision-making timeline and provide insights into the team dynamics, challenges, and areas of opportunity, particularly in relation to the factorial trial. We provide lessons learned that could be refined and tested in future work to contribute to the development of best practices on forming and maintaining MSPs between CBOs, collective impact coalitions, FQHCs, and academia.

## 2 Methods

### 2.1 Multi-sector partnership formation

The HEALthy4You multi-sector partnership was established in response to a funding opportunity from the California Initiative for the Advancement of Precision Medicine (CIAPM). Formed in September 2020, the initial grant writing team included UC San Diego (UCSD) faculty from public health, psychiatry, bioinformatics, and clinical and translational research. Early in the process, the original principal investigator transferred institutions, necessitating a change in leadership. An experienced senior investigator from UC San Diego's Altman Clinical and Translational Research Institute (ACTRI) volunteered to lead the initiative, engaging expertise from the ACTRI in trial methodology, human subject protections, implementation science, study coordination, sample processing, biobanking, and regulatory compliance.

With this leadership change, the team was restructured to invite multiple principal investigators and support from the San Diego County Childhood Obesity Initiative (SDCOI). SDCOI partners, including Poder Popular, Kitchenistas, and Comité Organizador Latino de City Heights (COLCH), were invited due to their work in obesity prevention and their grassroots connections in communities with FQHC clinics. Additional partners, including representatives from the American Academy of Pediatrics; Vista Community Clinic; Olivewood Gardens and Learning Center, San Diego State University; and Streetwyze, were noted as key collaborators, and other partners were designated as co-investigators or co-principal Investigators. Partners brought lived experience and assisted with study development and intervention materials. For example, informed consent forms were reviewed and edited by community members and were refined with community input before submission to the UC San Diego Institutional Review Board (IRB). Partners were compensated for their time and effort. Partners from Streetwyze helped to lead a component of the project focused on community and neighborhood-level data collection, which was intended to complement findings from the clinical factorial trial.

### 2.2 Grant proposal objectives

The grant was awarded in May 2021 with a start date of September 2021. As outlined within the grant proposal and aligned with the funder's explicit request for community engagement, three sectors [community (with support from SDCOI), academia, and clinical partners] worked collaboratively in the first year to design the precision population health approach. The team set forth preliminary priorities of implementing a family-centered program in primary care, focusing on Latino families, utilizing evidence-based strategies, and testing a program to address the experience of ACEs. The resulting study aimed to implement a multicomponent program that could be delivered within an FQHC in collaboration with the SDCOI to improve family resilience to ACEs and treat childhood obesity among Latinos. Family Health Centers of San Diego (FHCSD) was selected as the implementing FQHC. Notably, the grant was not initially selected as one of the funded studies, but the new PI worked with co-PIs, CIAPM, state government officials, and community partners to collectively discuss and advocate for funding, ultimately securing approval through the state budget in Governor Newsom's office.

### 2.3 Project framework

The team utilized a *co-creation* approach combined with the Exploration, Preparation, Implementation, and Sustainment (EPIS) framework (4, 8) to facilitate a collaborative design process. Co-creation involves activities and processes primarily led by community partners, builds on the strengths and resources of the community, promotes co-learning and mutually beneficial activities, and achieves a balance between research and action (4, 9). Combining co-creation approaches with implementation science can provide a comprehensive and rigorous organizational structure. The EPIS framework draws attention to outer and inner contextual factors that might inform implementation, as well as identifies variables that can influence implementation. Within EPIS, co-creation is a “bridging factor” necessitating collaboration among relevant parties in the ecosystem's outer and inner contexts to shape an innovation's adoption and scale (10). Bridging factors refer to relational ties, arrangements, and processes serving as the connective tissue across contexts (11). For this project, a significant amount of time was spent in the “exploration” and “preparation” phases, with checkpoints for feedback from relevant parties for iterative design. The application of the EPIS framework is discussed in more detail in the Results section.

### 2.4 Retrospective process evaluation

After study launch and recruitment began in January 2023, the team conducted a retrospective process evaluation in March 2023 to identify situational factors associated with project challenges and delays (see Table 1 for the study timeline), particularly relevant to the factorial trial. The goal was to uncover how and why challenges or delays occurred, with the hope that contributing factors could be identified and learnings might guide future MSPs between communities/coalitions, FQHCs, and academics. The process evaluation included three phases: (1) multi-rater document and meeting note coding (12), (2) a timeline review from study design to implementation, and (3) multi-rater evaluation using the Pragmatic–Explanatory Continuum Indicator Summary Rating System (PRECIS) to rank methods domains (e.g., recruitment, setting, and organization) along the pragmatic–explanatory continuum of the factorial trial (i.e., the primary aims of HEALthy4You).

**Table 1:** Files reviewed.

File type	Number of files
Word documents (meeting notes)	85
Email files	4
PDF files	10
Audio recordings	14
Total	115

### 2.5 Phase 1: thematic coding of study team documents

Two members of the UC San Diego research team organized study documents (*N* = 115) chronologically, including meeting minutes, action items, email chains, and audio recordings from the start of grant preparation to the launch of recruitment (see Table 1). Files dated back to November 2020, prior to grant submission; receipt of funding began in September 2021; and study launch was in January 2023. A reflexive approach in which researchers consider how their views and feelings have influenced findings was employed for coding notes and documents. This approach allows researchers to uncover “unexpected meanings rather than summarize the data [and] are interpreted through researcher's assumptions, commitments, and scholarly knowledge” (13). This coding process leads to a synthesis of themes reflecting patterns of shared meaning and understanding, which are explicitly embedded in the social context where the work was done. Thus, it is not meant to be an “objective” analysis but, instead, is guided toward reviewing with purpose and context to understand and reflect on dynamic issues of power and decision-making. The research team reviewed codes and developed a table to summarize qualitative themes that emerged from code review (Table 2).

**Table 2:** Key decisions and actions.

Key decision	Year 1												Year 2											
Exploration	09/21	10/21	11/21	12/21	1/22	02/22	03/22	04/22	05/22	06/22	07/22	08/22	09/22	10/22	11/22	12/22	01/23	02/23	03/23	04/23	05/23	06/23	07/23	08/23
Factorial trial																								
Sample size: *n* = 200 sample																								
Duration of study: 6 months																								
Interventions: adapted IYP and Promotora																								
Inclusion and exclusion criteria																								
Management team formation																								
Preparation																								
Hiring of intervention staff																								
Screening process (i.e., based on provider discretion)																								
Training protocols for wellness coach, promotoras, parenting trainers																								
Planned recruitment at four FHCSD clinics																								
Implementation																								
Study recruitment launch at FHCSD																								

IYP, incredible years program.

Note: Blue = year 1, green = year 2, light green = no decisions were occurring during these periods.

### 2.6 Phase 2: timeline review

The research team reviewed the chronologically organized study documents to develop a timeline demarcating key events and decisions made during the first and second years (Table 2). The timeline was also used to compare the expected month of study launch (September 2021) with the actual study launch (January 2023).

### 2.7 Phase 3: pragmatic–explanatory continuum indicator summary ratings

Two academic researcher team members independently rated the HEALthy4You study on each methods domain of the PRECIS model (14, 15), as presented in Table 3. The PRECIS-2 is a framework used by trialists to assess where their clinical trial design sits on the spectrum between a purely pragmatic approach (real-world effectiveness) and a purely explanatory approach (testing a mechanism under ideal conditions). The tool helped to evaluate where the study fell on the pragmatic–explanatory continuum and contextualize tensions identified through the document review. Each PRECIS domain (outcome, analysis, eligibility, etc.) includes a question to facilitate rating. For example, for the domain of primary outcome, the prompt is, “To what extent is the trial's primary outcome relevant to participants?”. The rating scale ranges from 1 very explanatory, 2 rather explanatory, 3 equally pragmatic–explanatory, 4 rather pragmatic, to 5 very pragmatic. Table 4 presents the average ratings of the two reviewers.

**Table 3:** Qualitative themes, subthemes, and excerpts.

Theme	Subtheme: underlying tension or question	Documented excerpt	Who are we missing?	Date
Ambiguity in defining study objectives and endpoints	What is the overarching objective of the trial?	“Is our goal to focus and reducing current trauma in families (with the hopes to reduce current trauma we will break the cycle of historical trauma, therefore can deduce would prevent trauma) or are we trying to prevent trauma in families who have parents with high ACE scores yet do not have documented current trauma?”	Community members not involved in the discussion	November 2020
	What is the scope of the study?	“If it needs to be really ACES-oriented (which means, either treat or prevent ACES traumas), then we did not land in a good place.”	Missing key operational FHC members and community members	December 2020
	Circular conversations around concepts, terms, and theoretical explanations; anticipating community involvement; timeline challenges	“What do we mean when we say trauma? Backing up—We should move forward to define an outcome. We should bring interventions/outcomes to the community for refinement but have ideas to start with else we’ll be talking about outcome, a, b or c for months on end.”	Community members not involved in the discussion	November 2021
	Co-primary measures; 2 × 2 factorial; timeline unclear/delays	“One thought that came to mind as I was doing this is that in the 2×2 factorial design, we are really testing 2 different interventions. Therefore, shouldn’t we be assessing each of those intervention components? I know we keep saying this, but I think we are close to being able to start writing this IRB. I think now we need to figure out some logistics of how all this will be put together, how will we measure all the different implementation components [at the external community, internal community (FHCs), and family level], and what will be required to make this work.”	Not involving key operational FHC members and community members	November 2021
	Mismatch between pressures faced by FQHC and their priorities, and the desired research focus of the academic team.	“If we don’t consider incidental to clinical care then we need an auditing trail to that lane.” “Depends on framing. In the general frame, can we say this is QI research? But we are still looking to get generalizable information for the grant?”	Missing additional FHC members and PI consensus/direction	January 2022
Need for increased flexibility for including clinical partners	Critical conversation without appropriate clinical stakeholder involvement	“Are these the collection measures we are using to test biological outcomes? In our last meeting we discussed using the stress cortisol testing as a more feasible measure as opposed to BMI.”	Not involving key operational FHC members and community members	November 2020
	Early intentions for a high level of community involvement	“That really draws us to linking with clinics early-on so that Promotoras know how and to whom they would be passing information on to.”	Missing key FHC members and community members	December 2020
	Early intentions for a high level of community involvement	“What about the community review? Should we focus more on getting input on interventions rather than outcomes?”	Community members not involved in the discussion	November 2021
	Early intentions for a high level of community involvement	“Need to have a few working meetings including the community level people, with the FHC people, and the design people to really ensure that everyone is heard and all needs/practicalities addressed in a more timely manner. If we can do this once or twice in Dec, then I think we could write this IRB in Jan.	Not involving key operational FHC members and community members	November 2021
	Critical conversation without adequate clinical and community stakeholder involvement	Provide parenting training to 2 of the 4 promotores so they can reinforce skills with parents during weekly check-ins.”	Not involving key operational FHC members and community members	February 2022
Differing perspectives among team members on research approaches	Importance of pragmatism vs. explanatory research	“The question for sustainment, in my opinion, is the degree to which a community-centered learning healthcare system could be supported without grant funding.”	Missing key operational FHC members	December 2020
	Clarification of the type of research—quality improvement vs. clinical trial	“What is the focus of the trial? Improve patient-reported outcomes relevant to traumas via promoting trauma-responsive relationships.” “Gather data around implementation and adaptation of evidence-based interventions and approach within FHC. Meeting Slides”	No consensus decision-making/maker	October 2021
	Challenges with precision matching and factorial trial	“I am a little worried about Factorial Design and Micro-randomization because it can get really complicated with all the different intervention components. It could also get really complicated because the characteristics of the families who enter this program are going to be REALLY diverse.”	Not involving key operational FHC members and community members	November 2021
	Intention to be rigorous and control timing	“We need to figure out the pacing of these visits and when we want to send to Healthy Together. Meeting Minutes.”	Missing key FHC members	December 2021
	Framing as quality improvement research in the FHCSD context is in tension with clinical trial objectives	“Need to separate activities within the project that are related to care/quality improvement, to activities that are pure research (e.g., biobanking, qualitative surveys, Streetwyze utilization by families, etc.). Meeting Minutes”	Not involving community members and key operational FHC members	January 2022
	Importance of pragmatic intervention	“Important to integrate the interventions within the clinical care as much as possible, and reduce the “extra stuff” in order to reduce the burden on the families.”	Not involving key operational FHC members	January 2022
Hurdles operationalizing research workflows	Rigorous planning without practical implementation knowledge; pacing of sessions and HT; needed to learn by doing	“We need to determine how many sessions/meetings we want to have right off the bat to make sure it’s not too much of a burden on families.”	Not involving key operational FHC members	January 2022
	Rigorous planning without practical implementation knowledge	“Staged launch, site by site; What is steady state of intervention about 40 families in each condition.”	Not involving key operational FHC members	January 2022
	Assumptions made about clinical operations and workflow that were not feasible	“We should write script for PCP about the program If comfortable with learning more, MA joins visit (warm hand-off) for meet and greet and explains the program. If not time or not sure about it, WC will follow up by phone within the next few days to try to get them in the program.”	Not involving key operational FHC members	February 2022
	Recruitment considerations; without considering practical and feasibility	“Can we write script for PCP about the program If comfortable with learning more, MA joins visit (warm hand-off) for meet and greet and explains the program. If not time or not sure about it, WC will follow up by phone within the next few days to try to get them in the program.”	Not involving key operational FHC members and community members	February 2022
	Organizational tensions/bureaucratic barriers	“We need to present the hypothesis of the biomarkers in a table, indicating each biomarker, why is useful, citations to back that up, implications for results, pathways if abnormal results. The CMO needs to approve it. And make sure there are no hidden costs for the clinic or extra burdens for the families.”	Missing key FHC members and community members	March 2022

PCP, primary care provider.

**Table 4:** Pragmatic explanatory continuum indicator summary ratings.

PRECIS domain	PRECIS design question	PRECIS rating^a^	Proposed design	Challenge/tensions with design decision	Final operational decision
Primary outcome	To what extent is the trial's primary outcome relevant to participants?	4	BMI (childhood obesity)	We shifted to address underlying drivers of obesity and ACEs through intervening on social factors. Addressing clinical disease (i.e., obesity) associated with ACEs will increase the likelihood of observing treatment effects in a short 6-month timeframe. Also, FHCSD had an existing multicomponent program called HealthyTogether addressing childhood obesity that needed resource investment and revamp, so there was an opportunity to intervene within the context of the HealthyTogether program	Parents’ assessment of protective factors as primary outcome
Primary analysis	To what extent are all data included in the analysis of the primary outcome?	3	Include all intent-to-treat data in analysis	None	Analysis will be conducted as both intention to treat and per protocol to examine dosage effects
Eligibility	To what extent are the participants in the trial similar to those who would receive this intervention if it were part of usual care?	4	Children, aged 5–11, with>1 ACEs identified on PEARLS assessment	FHCSD wanted the fewest criteria to recruit the highest number of families and to have evidence for expanding the program to other ethnicities; UCSD wanted to have some criteria to examine the impact on a specific population subset with the greatest likelihood of program benefit. FHCSD does not currently routinely screen for ACEs for ages 5–11	Children, aged 5–11, identified as Latino, some exclusions for severe mental illness and developmental delay, Physician discretion for referral
Recruitment	How much extra effort is made to recruit participants over and above what would be used in the usual care setting to engage with patients?	5	Physician referral/warm hand-off to wellness coach	FHCSD constraints with clinic space, and not as many potentially eligible participants are scheduled for well child visits	Implement a different recruitment strategy such as cold calling of potential participants
Setting	How different is the setting of the trial and the usual care setting?	5	Utilize existing primary care space at FHCSD for delivery of intervention components	FHCSD does not have sufficient space to accommodate weekly Promotora intervention sessions required for H4Y trial	Remote and telehealth appointments for promotoras and wellness coach
Organization	How different are the resources, provider expertise, and the organization of care delivery in the intervention arm of the trial and those available in usual care?	4	Utilize existing staff at FHCSD for delivery of intervention components including promotora intervention, Incredible Years parenting training, and wellness coach check-ins	FHCSD did not have sufficient staff resources to accommodate the interventions required for the H4Y trial	Research funds needed to support promotoras, wellness coach
Flexibility (delivery)	How flexible is intervention delivery as compared with usual care?	5	Quality improvement research	FHCSD wanted the program to operate as a quality improvement to maximize implementation, sustainability, and net benefit for the institution However, this project was funded by the California Institute for the Advancement of Precision Medicine as a Clinical Trial	Pragmatic factorial trial with aspects of implementation science and focus on sustainability
Flexibility (adherence):	How different is the flexibility in how participants must adhere to the intervention, and the flexibility likely in usual care?	5	The promotora intervention is designed to be at least one check-in/session per week. The Incredible Years intervention is one session every week for the first month, and then biweekly until 12 sessions	The additional sessions may be burdensome to families	
Follow-up	How different is the intensity of measurement and follow-up of participants in the trial and the likely follow-up in usual care?	3	Surveys at baseline and 3 and 6 months and to collect them as part of routine clinical care as opposed to introducing extra research surveys	To align with quality improvement goals, we were hoping to rely on existing clinical instruments or tools for research measurement. However, we are interested in variables like child attachment, child self-regulation, and family social support, which are not routinely collected in clinical care	Surveys at baseline and 3 and 6 months would be collected by phone, text, or email by research assistants BMI collection at baseline and 3 and 6 months by physician
	Mean	4.2			

^a^ Average ratings across two reviewers; Scale is from 1 (highly pragmatic) to 5 (highly explanatory).

### 2.8 Phase 4: timeline of team engagement

To systematically document stakeholder engagement during the HEALthy4You startup phase, we developed the HEALthy4You Timeline of Team Engagement in Table 5. The table charts stakeholder involvement across academic, clinical, and community sectors during the 24-month startup phase, mapping engagement levels using the “Spectrum of Public Participation” (16). Engagement levels were inform (I), consult (C), participate (P), initiate (In), and lead (L), representing increasing levels of influence on decision-making processes. Research assistants reviewed team notes, meeting agendas, and participant attendee lists to categorize team member engagement in team decision-making. The table was reviewed and discussed by the co-authors.

**Table 5:** HEALthy4You timeline of team engagement.

MSP Sector	Year 1												Year 2											
Academia	09/21	10/21	11/21	12/21	1/22	02/22	03/22	04/22	05/22	06/22	07/22	08/22	09/22	10/22	11/22	12/22	01/23	02/23	03/23	04/23	05/23	06/23	07/23	08/23
UCSD clinical and translational research expert	L	L	L	L	L	L	C	C	C	C	C	C	C	C	C	C	C	C	C	C	C	C	C	C
UCSD public health professor	L	L	L	L	L	L	L	L	L	L	L	L	L	L	L	L	L	L	L	L	L	L	L	L
UCSD executive director of center for community health	C	C	C	C	C	C	C	C	C	C	C	C												
UCSD dissemination and implementation science expert	C	C	C	C	C	C	C	C	C	C	C	C	I	I	I	I	I	I	I	I	I	I	I	I
UCSD pediatrician researcher	C	C	C	C	C	C	C	C	C	C	C	C	C	C	C	In	In	In	In	In	In	In	In	In
SDSU public health professor	C	C	C	C	C	C	I	I	I	I	I	I	I	I	I									
UCSD medical informatics expert	C	C	C	C	C	C	C	C	C	C	C	C	C	C	C	C	C							
UCSD biostatistician	C	C	C																					
UCSD biospecimens and biobanking expert						C	C	C	C	C	C	C	C	C	C	C	C	C	C	C	C	C	C	C
UCSD/SDSU graduate student researcher	P	P	P	P	P	P	P	P	P	P	P	P	P	P	P	P	P	P	P	P	P	P	P	P
UCSD project management team	In	In	In	In	In	In	In	In	In	In	In	In	In	In	In	In	In	In	In	In	In	In	In	In
UCSD community lead	In	In	In	In	In	In	In	In	In	In	In	In	In	In	In	In	In	In	In	In	In	In	In	In
Clinical																								
FHCSD research clinic director	L	L	L	L	L	L	L	L	In	In	In	In	C	C	C	C	C	C	C	C	C	C	C	C
FHCSD wellness coach											C	C	C	C	C	C	C	C	C	C	C	C	C	C
FHCSD promotoras													C	C	C	C	C	I	I	I	I	I	I	C
FHCSD mental health clinicians												C	C	C	C	C	C	I	I	I	I	I	I	C
FHCSD PCP providers												C	C	C	C	C	C	I	I	I	I	I	I	C
Community																								
American Academy of Pediatrics, CA Chapter 3 (AAP-CA3)	C	C	C	C	C	P	P	P	P	P	C	C	I	I	I	I	I	I	I	I	I	I		
Streetwyze	C	C	C	C	L	In	In	In	P	P	P	P	P	P	P	P	P	P	P	P	P	P	P	P
Kitchenistas of Olivewood Gardens and Learning Center	**I**	**I**	C	C	C	C	C	P	P	P	P	P	P											
Poder Popular of Vista Community Clinic	**I**	**I**	C	C	C	C									C	C	P	P	P	P	P			
County Obesity Initative Tri-Chair/SD Promotores (Margarita Holguin)	C	C	C	C	C	P	P	P	P	P	P	P	P	P	P	P	P	P	P	P	P	P	P	P
Comité Organizador Latino de City Heights (COLCH)	C	C	C														C	C	P	P	P	P	P	
Partners in Prevention						**C**																		
South Bay Community Services								C	C	P	P	P	P											
Chicano Federation									C	C	P	P	P	P	P	P								

L, lead; C, consult; I, engagement levels were inform; In, initiate; P, participate.

Note: Blue = year 1, green = year 2, light green = no decisions were occurring during these periods.

## 3 Results

### 3.1 Application of the project framework

During the EPIS “exploration” phase, four teams set up weekly meetings (i.e., measures, operations, community, and intervention). This phase involved meeting with different team members and community partners to review a range of evidence-based interventions that might suit the context and proposed aims. The “preparation” phase involved development and refinement of the fidelity and adaptation monitoring process by creating the framework by which the intervention could be deployed and assessed throughout the subsequent “implementation” and “sustainment” phases. These latter phases had only just begun at the time of the process evaluation and will be evaluated in future studies.

### 3.2 Team structure and process

Due to the COVID pandemic, the team met and interacted virtually using Zoom™, email, Slack™, and Google Workspace. Initially, the team met biweekly as a full team with FHCSD, community leaders (including a master trainer of promotoras and a lead in child and family care community interventions), community representatives from Vista Community Clinic and Olivewood Gardens, and UC San Diego representatives from September 2021 to December 2021. Starting January 2022, the research team was divided into four teams for weekly meetings (i.e., operations, measures, community, and intervention) to streamline project discussions and decision-making and to discuss various aspects of project design. The “operations team” included project management, principal investigators, and research staff. The “measures team” included experts in methods, behavioral science, implementation science, quantitative analytics, support staff, and our lead FHCSD partner with expertise in pragmatic implementation studies. The “community team” included principal and co-investigators (including lead investigators representing Vista Community Clinic), representatives of partners from local organizations (including the SDCOI, Streetwyze, FHCSD, COLCH, and Olivewood Gardens). It should be noted that the community team focused more deeply on the environmental and policy interventions that were enacted by the SDCOI and Streetwyze. These activities, while part of the overall project, are not the focus of this present review. The “intervention team” included investigators, research and clinical experts in pediatric interventions and implementation science; an expert in the community health worker (CHW) field, a representative from the American Academy of Pediatrics, CA Chapter 3 (AAP-CA3); and community partner representatives such as promotoras.

### 3.3 Decision-making timeline

Table 1 displays the approximate durations of key team decisions from the start of the study period (receipt of grant funding in September 2021) through the end of the study period (end of the second year in August 2023). Decisions related to study design, sample size, and study duration required 3 or fewer months. Intervention development, recruitment processes and procedures, sample characteristics, training protocols, and intervention components decisions took at least 9 months. With regard to team structure, it took 13 months to solidify the management team, including the hiring and training of a full-time program manager and 12 months to recruit and hire staff through the clinical partner organization. Compared with early timeline estimates noted in team documentation, the final intervention components were delayed by 7 months from the initial timeline, while finalizing recruitment processes and study launch were delayed 8 and 4 months, respectively. Delays were, in part, due to complexities with hiring through FHCSD, challenges with subaward disbursement, sponsoring research staff who reside in Tijuana (Mexico) but work in San Diego, and human resource delays at UCSD.

### 3.4 Qualitative themes

Four themes emerged from documentation review and coding: (1) ambiguity in defining study objectives and endpoints, (2) hurdles operationalizing research workflows, (3) need for increased flexibility for including clinical partners, and (4) differing perspectives among team members on research approaches (e.g., pragmatic, explanatory, and precision approaches). Table 2 displays the themes with example excerpts.

### 3.5 Theme 1—ambiguity in defining study objectives and endpoints

The theme “ambiguity in defining study objectives and endpoints” involved notable subthemes: (1) redundant discussions around objectives, aims, frameworks, samples, and outcomes; (2) reconsideration of primary outcomes; and (3) establishing the scope of the research. For example, one team member in November 2021 encouraged the team to redefine terminology and clarify outcomes, “What do we mean when we say trauma? Backing up—We should move forward to define an outcome. We should bring interventions/outcomes to the community for refinement but have ideas to start with or else we’ll be talking about outcome, a, b or c for months on end.” Team documentation also revealed an order of operations that was often repetitious. The omnipresent influence of timelines required for the clinical trial and complexities of managing budgets challenged the team's efficiency, especially when the protocol, measures, and processes were often in flux. For example, the team held conversations to finalize training processes for personnel ahead of finalizing intervention components.

### 3.6 Theme 2—hurdles operationalizing research workflows

The theme “hurdles operationalizing research workflows” refers to the challenges and delays in outlining the study's logistical workflows. Early team meetings were focused on ideating and theorizing, with few decisions on methods and operations. Issues related to the feasibility within clinical operations and workflow often challenge progress and research plans. For example, the team initially planned for providers to screen potential participants for ACEs using a documented score on the State-approved PEARLS tool. However, this was impractical within clinical processes due to limited healthcare provider time and clinic staffing. Organizational constraints also made working with clinical staff challenging due to their limited bandwidth, staff productivity targets, and risk aversion (e.g., requesting a supplemental insurance policy for collecting blood for research at the time of clinical blood draw). Planning discussions took place without a full appreciation of the clinic workflow and what might be appropriate for the local context. For example, meeting minutes from February 2022 revealed that team members discussed how to operationalize recruitment in the clinic without considering the burdens on the medical assistants (MAs) and primary care providers (PCPs) to involve them in this way: “We should write a script for PCPs about the program. If comfortable with learning more, MA joins visit (warm hand-off) for meet and greet and explains the program. If [there is] not time or [patient is] not sure about it, wellness coach will follow up by phone within the next few days to try to get them in the program.”

### 3.7 Theme 3—need for increased flexibility for including clinical partners

The theme “need for increased flexibility for including clinical partners” reflects that the project would have benefited from greater involvement from relevant clinical partners (e.g., patients, providers, and those directly involved in implementation and workflow), but was unable to flexibly engage providers in the ways that would align with their priorities, schedules, and staffing constraints. Emergent subthemes included (1) early intentions for a high level of community and clinic partner involvement and (2) decision-making without relevant FHCSD clinic and patient stakeholders. For example, the first meeting with mental health providers, a key partner for implementation, did not occur until approximately 12 months after funding commenced. At times, assumptions regarding current processes and barriers seemed to be made without soliciting critical community and clinical members’ feedback. In documentation from November 2021, one member underscored the need to ensure relevant partner involvement to accelerate decision-making: “We need to have a few working meetings including the community level people, with the [FHCSD clinical] people, and the design people to really ensure that everyone is heard and all needs/practicalities addressed in a more timely manner.” However, incorporating clinical members’ feedback within a strict timeframe and rigid interpretation of what was originally proposed without clinical members’ input proved challenging for the team.

### 3.8 Theme 4—differing perspectives among team members on research approaches

Divergent research approaches and perspectives from team members led to delays in design and implementation. Salient subthemes included (1) desire for pragmatic vs. explanatory (i.e., highly controlled) research, (2) framing of the study as quality improvement potentially conflicting with the goal of generalizable knowledge and regulatory requirements for human subject research, and (3) differing theoretical approaches and understandings of what “precision matching” meant within the trial, an important component and priority of the funded proposal. For example, documentation from January 2022 showed an attempt to untangle components considered quality improvement vs. research: “We need to separate activities within the project that are related to care/quality improvement from activities that are pure research (e.g., biobanking, qualitative surveys, Streetwyze utilization by families, etc.).” Others emphasized pragmatism: “It's important to integrate the interventions within the clinical care as much as possible and reduce the ‘extra stuff’ to reduce the burden on the families.” These conversations seemed to loop without resolution, revisiting the same points without reaching a clear consensus.

### 3.9 PRECIS ratings

The average PRECIS rating across reviewers and domains was 4.2, defining the trial as *rather pragmatic.* The domains of primary analysis and follow-up were assigned the lowest rating of 3, defining the trial as *equally pragmatic–explanatory*. The domains of flexibility (delivery), flexibility (adherence), setting, and recruitment were assigned ratings of 5, *very pragmatic*. None of the domains received a *purely explanatory* rating of 1, nor a *rather explanatory* rating of 2. The average PRECIS ratings of the two reviewers are listed in Table 4.

### 3.10 PRECIS ratings

The average PRECIS rating across reviewers and domains was 4.2, defining the trial as *rather pragmatic.* The domains of primary analysis and follow-up were assigned the lowest rating of 3, defining the trial as *equally pragmatic–explanatory*. The domains of flexibility (delivery), flexibility (adherence), setting, and recruitment were assigned ratings of 5, *very pragmatic*. None of the domains received a *purely explanatory* rating of 1, nor a *rather explanatory* rating of 2. The average PRECIS ratings of the two reviewers are listed in Table 4.

### 3.11 HEALthy4You timeline of team engagement

Academic experts maintained leadership (L) and consultative (C) roles across the 24-month startup phase, with project managers serving in participatory (P) roles. In the clinical sector, five frontline healthcare professionals, such as nurses and primary care providers, were involved, but only one held a leadership (L) role during the study's first 8 months, with the remaining clinical stakeholders transitioning to consultative (C) or participatory (P) roles. In the community sector, seven key community partners were involved, but only two sustained a participatory (P) role throughout, with three other community representatives showing fluctuating levels of engagement.

## 4 Discussion

MSPs are increasingly seen as central for advancing health for everyone, everywhere (4), yet there is little evidence to guide operationalizing these collaborative partnerships. Further, there is a need for MSP best practices in research, especially as funders such as the NIH and PCORI expand investment in such models. This paper presents a reflective process evaluation of co-creating and launching the HEALthy4You study in an FQHC in partnership with the community and academics, and describes project tensions and areas where greater attention is needed to facilitate efficiency and reduce redundancy. Our process of identifying and resolving these tensions between partners across multiple levels prompted the following five lessons:

### 4.1 Lessons learned

#### 4.1.1 Lesson 1—define project goals and priorities early on

Ambiguity and competition between priorities, in terms of both theoretical approaches (pragmatism vs. explanatory) and implementation goals, can produce inefficiencies and delays in operational activities. In our case, these delays were compounded by institutional protocols, such as obtaining IRB approval and meeting requisite grant deadlines. Greater flexibility is needed by funding agencies regarding changes to study timelines and deliverables. These challenges can be ameliorated by explicitly defining project goals and priorities before the proposal goes in and routinely addressing questions such as “What are the overarching objectives of this study?” and “What is the practicality or feasibility of implementing this intervention or component within the organizational setting?” early in the exploration phase. Establishing mutually agreed-upon high-level goals early can facilitate shared understanding and motivation among a broad team.

#### 4.1.2 Lesson 2—ensure partners are involved from the outset and are well-represented at meetings to facilitate shared understanding

Andress et al. (17) found that community-academic partnerships only address power dynamics or differences in rank, privilege, or power when they become issues from the community's perspective. Fully addressing Lesson 1 requires that all relevant partners, including clinical and community stakeholders, discuss power and decision-making from the outset of the exploration phase, especially during project conceptualization and grant submission. The International Association of Public Participation Community Engagement Continuum (18) can help teams reflect on the extent to which power is equitably shared among partners and operations are participatory, helping teams move from “consulting” and “involving” to authentic “shared leadership.” This is consistent with commonly articulated best practices in CBPR such as fully partnering with community leaders in the research process and ensuring research is co-led by community partners. While the goal was to be community-centered and co-developed, in practice, these activities were primarily driven by the academic PIs. The academic partners took a stronger driving role than may be warranted (although understanding of the right type of leadership is contingent upon a clear understanding of goals, see Lesson 1).

#### 4.1.3 Lesson 3—co-develop shared power and decision-making structures between community partners and researchers to ensure community voice in planning and implementation

Capitalizing on the successful inclusion of all key research, administrative, clinical, and community partners through Lesson 2 requires formal recognition of decision-making pathways. Shared governance is increasingly being recognized as important, and studies are encouraged to articulate approaches to actualize shared leadership between researchers, who bring scientific knowledge and domain expertise, and clinical and community members, who bring real-world knowledge on what is practical, possible, and most important (19). This ensures that all partners are not only represented but equipped with sufficient power to inform key decisions throughout EPIS phases and for bridging outer and inner contexts.

#### 4.1.4 Lesson 4—conduct research readiness and capacity assessment to identify potential barriers and inform study planning

It would be prudent to understand current limitations in the EPIS outer context, inner context, and bridging factors. For example, when integrating research processes and new interventions into a new organizational setting (inner context), it is important to fully understand staff perspectives (e.g., healthcare providers and clinical administrators) and leadership perspectives (e.g., investigators and research leads) to make interventions viable and sustainable. We found that these perspectives can be divergent. By comprehensively assessing barriers and capacities early on during exploration, partners and investigators can, in turn, develop actionable strategies for addressing and mitigating challenges that may arise, leading to smoother implementation.

#### 4.1.5 Lesson 5—implement iterative design and testing to learn by “doing”

Building in ample time for addressing EPIS factors can be facilitated through engaging in iterative design, formative testing, and development. For example, conducting iterative proof-of-concept studies or Plan-Do-Study Act (PDSA) cycles prior to and during implementation could help facilitate learning about the practical implementation barriers and resource constraints ahead of study launch (20). Similar to PDSA, several rapid cycle learning models exist in the fields of improvement science and quality improvement to guide implementation efforts in healthcare settings and to provide a structure for rapid experiential learning in real-world settings (20–22). Establishing relevant quality improvement or clinical benchmarks through proof-of-concept or PDSA trials may also be necessary for justifying sustainment and useful in advocating for continued clinic investment (e.g., patient satisfaction, attendance, cost effectiveness, and trust in providers), although studying clinical effectiveness may be most important to funders. Mainly, early operationalization during “exploration” with time to iteratively refine and improve is critical for “implementation” and “sustainment.”

### 4.2 Practical implications

Increasing investment from funders such as the PCORI, NIH, and RWJF underscores the urgency of building models that meaningfully share power. As noted, NIH's ComPASS Program, for example, exemplifies this shift by scaling community-led health equity interventions through MSPs to tackle persistent disparities. For those that are newly funded by ComPASS and similar programs, integrating these lessons into their research from the outset is not only important for enhancing feasibility but also for ensuring that community partnerships remain sustainable. Embedding power-sharing and equity at the core of design strengthens trust, improves relevance, and increases the likelihood that solutions will endure and meaningfully improve health in marginalized communities.

These five lessons also build on a growing evidence base of frameworks for community engagement and participatory research (23–25). For example, Participation choice points in the research process (26) can be helpful for identifying who should be participating in key decision-making processes and when. MSP teams must think critically about how often meetings are needed; the format, location, and structure of meetings; and who needs to be present to ensure implementable decisions are made. Thoughtfully addressing who is at the proverbial “table” (i.e., who needs to be present to think about intervention implementation and sustainment) can facilitate early identification of potential conflicts in goals and priorities and promote consensus prior to advancing into the “preparation,” “implementation,” and “sustainment” phases, when changing course can be more challenging. In our case, our project may have struggled with efficient decision-making due to turnover among staff and community partners. Community representatives from COLCH, potential participant families, and relevant FQHC clinical staff and decision-makers could have been more fully integrated into meetings and decision-making pathways from the outset with remuneration for their time.

Gaps were made visible in the Healthy4You Timeline of Team Engagement as community partners were rarely in decision-making roles, and participation fluctuated over time. These underrepresented voices limited the diversity of perspectives and may have contributed to challenges with acceptability and feasibility. Inviting program officers, funders, policymakers, and senior leadership across organizations to regular meetings and progress updates may also strengthen sustainability and help to align evidence with policy action. Identifying, including, and empowering all partners directly promotes improved understanding of the “implementation” and “sustainment” landscape, allowing for early solidification of project objectives with equitable input, interpretation, and agreement from all partners. This might also entail building trust and creating relationships with several clinical and community partner representatives to buffer against job turnover, coupled with a budget to support adequate involvement. These relationships can help keep attention toward “sustainment,” rather than the outcomes of a singular study.

Reaching consensus on where the study falls on the spectrum of pragmatism and explanatory is also important for guiding prioritization of goals and downstream decision-making. Our focus on pragmatism and delivering practices within routine care to a highly diverse sample may have inadvertently allowed for so much flexibility that it might be difficult for us to see health improvement outcomes important for decision-makers, although this is an empirical question we will be able to explore when trial analysis is completed (dated for October 2025). Simultaneously, continuing an implementation study without real-time adjustments to the protocol (to heed to more rigorous randomized controlled trial (RCT) standards) may narrow the implementation information and outcomes that can be gleaned to inform real-world practicality. Identifying these tradeoffs during the “exploration” and “preparation” phases may inform decisions as the project advances into “implementation” and “sustainment.”

### 4.3 Limitations

Our results might be limited by biases among internal staff. Possible biases include observer bias in our team reporting on notes, coder confirmation bias in looking to validate a specific theme or themes, and/or inadvertently ignoring possible themes from other perspectives. Biases may have been reduced with coding of team transcripts by multiple external reviewers, which we did not have the capacity for in this process evaluation.

## 5 Conclusions

This article presents a case study of an MSP in San Diego County with resultant lessons learned. There is growing interest and investment in innovative funding models for MSP. The hypothesized best practices may be applied to community-engaged research trials facilitated by MSPs, especially when funding mechanisms allow for sufficient time for co-creation and partnership building: (1) conducting organizational readiness and capacity assessments; (2) defining project goals and priorities early; (3) involving all relevant partners, including clinical and community partners; (4) co-developing shared power and decision-making structures; and (5) employing iterative design and testing to address practical implementation barriers and resource constraints. Moving forward, we need models that truly support community leadership, such as co-developed governance structures, decision-making authority for community members, and flexible funding to address community priorities. These shifts can help move partnerships beyond a researcher-led model toward more balanced, sustainable collaboration.

## Data Availability

The raw data supporting the conclusions of this article will be made available by the authors, without undue reservation.
